# Sublobar Resection With Adequate Margin is Comparable to Lobectomy in Locoregional Recurrence

**DOI:** 10.1093/icvts/ivag045

**Published:** 2026-02-10

**Authors:** Megumi Nishikubo, Tappei Shomoto, Sanae Kuroda, Yuki Nishioka, Nahoko Shimizu, Wataru Nishio

**Affiliations:** Division of Chest Surgery, Hyogo Cancer Center, Akashi, Hyogo 673-8558, Japan; Division of Chest Surgery, Hyogo Cancer Center, Akashi, Hyogo 673-8558, Japan; Division of Chest Surgery, Hyogo Cancer Center, Akashi, Hyogo 673-8558, Japan; Division of Chest Surgery, Hyogo Cancer Center, Akashi, Hyogo 673-8558, Japan; Division of Chest Surgery, Hyogo Cancer Center, Akashi, Hyogo 673-8558, Japan; Division of Chest Surgery, Hyogo Cancer Center, Akashi, Hyogo 673-8558, Japan

**Keywords:** non-small-cell lung cancer, surgery, locoregional recurrence, margin distance, sublobar resection

## Abstract

**Objectives:**

Although sublobar resection is a standard treatment for small peripheral non-small-cell lung cancer (NSCLC), the optimal margin distance remains under investigation. This study aimed to determine the adequacy of commonly used margin distances by comparing locoregional recurrence (LRR) with lobectomy.

**Methods:**

We retrospectively reviewed data from patients with completely resected ≤3 cm adenocarcinoma, squamous cell carcinoma, or adenosquamous cell carcinoma treated between April 2018 and March 2024. We compared sufficient and insufficient margin sublobar resection and lobectomy in terms of freedom from LRR (FLRR) rate and the cumulative risk of LRR.

**Results:**

Of the 528 included patients, 200 underwent sublobar resection and 328 underwent lobectomy. After excluding 23 patients with pure ground-glass nodules, 505 patients with 19 LRR events were included in the prognostic analysis. In the overall cohort, LRR risk was worse in the insufficient margin group than in the sufficient margin and lobectomy groups, whereas the risk in the sufficient margin group was similar to the lobectomy groups (5-year FLRR: 75.1% vs 95.9% vs 95.8%, respectively). Conversely, in the pure-solid cohort, the risk of LRR was worse in the insufficient margin group and even in the sufficient margin group, compared to the lobectomy group.

**Conclusions:**

Among patients with completely resected ≤3 cm NSCLC, our study indicated that those who underwent sublobar resection with a sufficient margin achieved better local control than those with an insufficient margin, and sublobar resection with a sufficient margin was comparable to lobectomy. However, for pure-solid nodules, conventional margin distance might be insufficient.

**Clinical registration number:**

UMIN Clinical Trials Registry: Registration number: UMIN000058449. https://center6.umin.ac.jp/cgi-bin/ctr/ctr_view_reg.cgi?recptno=R000066821.

## Graphical abstract

**Figure:**
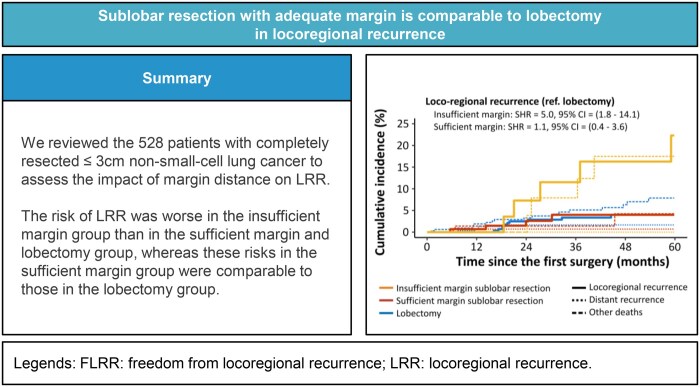

## INTRODUCTION

Based on recent randomized controlled trials, sublobar resection has become a standard treatment for small peripheral non-small-cell lung cancer (NSCLC).^1^^,^^2^ Nevertheless, the incidence of locoregional recurrence (LRR) after sublobar resection has been higher than that after lobectomy. Considering this fact, reducing LRRs could further improve the prognosis of patients undergoing sublobar resection. Although the National Comprehensive Cancer Network guidelines recommend that “sublobar resection should achieve parenchymal resection margins ≥2 cm or greater than or equal to the size of the nodule”,^3^ there is a scarcity of firm and high-quality evidence.

Consequently, several studies are attempting to decide an adequate margin distance,^4^^,^^5^ wherein the optimal margin distances were similar to the conventional threshold “parenchymal resection margins ≥2 cm or greater than or equal to the size of the nodule”. This cut-off has been used over the years; however, its adequacy has not been investigated, particularly in terms of comparison with lobectomy. In the above-mentioned previous studies, the validation analysis compared prognosis between the sufficient margin group and insufficient margin group; however, it is obvious that there exists a difference between them. LRRs would occur with a certain probability even when undergoing lobectomy. Therefore, the true goal is to reduce LRRs to the same level as those of lobectomy. In other words, it is important to avoid preventable LRRs by performing lobectomy.

In this study, we retrospectively compared the frequency of LRRs between patients who underwent sufficient margin sublobar resection, insufficient margin sublobar resection and lobectomy to explore whether the margin distance commonly used for sublobar resection is sufficient.

## PATIENTS AND METHODS

### Ethical statement

The study protocol complied with the Declaration of Helsinki and was approved on December 23, 2024, by the Clinical Research Area Ethics Committee of Hyogo Cancer Center (Registration No. G-428). As this was an observational study, the requirement for written informed consent was waived by the Institutional Review Board. This study was registered with the UMIN Clinical Trials Registry (UMIN000058449).

### Study design and patient selection

We reviewed the medical records of patients who underwent complete resection for ≤3 cm adenocarcinoma, squamous cell carcinoma, or adenosquamous cell carcinoma based on the previous studies^4^^,^^5^ at Hyogo Cancer Center between April 2018 and March 2024. Patients who received induction therapy; those who underwent combined resection of chest wall and those with pathologically confirmed lymph node metastasis, intrapulmonary metastasis, interlobular pl3 invasion, multiple lung cancers, or unreported margin distances were excluded. In our institution, surgical margin distances have been measured since 2018; therefore, we included all patients within this period.

The primary outcome was freedom from LRR (FLRR). The secondary outcome was cumulative incidence of LRR, recurrence-free survival (RFS), and overall survival (OS). Moreover, to speculate the adequate margin distance for each type of tumour, a scatter plot was generated.

### Surgical procedure

Pulmonary resection was conducted using video-assisted thoracic surgery (VATS) or thoracotomy. Standard complete VATS was performed with 1- to 4-port incisions, with a maximum size of 3.0-4.0 cm. Cases in which the incision was longer than 8.0 cm or rib resection was performed were defined as thoracotomies. Sublobar resections included wedge resections and segmentectomies. In most patients who underwent wedge resection, the tumours were detected by palpation and resected using mechanical stapler devices. For segmentectomy, all patients underwent contrast-enhanced CT unless there were contraindications to contrast agents, and images were generated through 3D reconstruction. After resection of the relevant vessels and the bronchus, we used a systemic injection of indocyanine green (0.3 mg/kg) to identify the demarcation line. When the tumour was located close to the intersegmental plane, the intersegmental veins were sacrificed, and the dissection line was set slightly shifted to the residual segment to secure the tumour margin. Preoperative CT-guided marking with a hook wire was used if pulmonary nodules were expected to be difficult to detect during the surgery.

### Measurement of margin distance

Pathologists measured the margin distance on formalin-fixed tissues after the stapled line was cut by the surgeon. Data were compiled by merging the shortest distance from the tumour edge, including the non-invasive component, to the resection margin with the width of the stapler (5 mm). The margin distance-to-solid component size ratio was defined as follows: margin distance-to-solid component size ratio = margin distance/tumour invasive size. Cases securing more than the invasive size of the nodule were defined as sufficient margin.

### Patient characterization

Clinical data were collected from the medical records at Hyogo Cancer Center. Pathological tumour stages were classified according to the Union for International Cancer Control classification, 8th edition. The observational follow-up to check for tumour recurrence and determine survival was conducted according the routine method of our institution. Ordinarily, we continue the observational follow-up for at least 5 years. During the first 2 years after the surgical intervention, systemic and local examinations are performed every 6 months, including blood tests, chest and abdominal CT, head MRI, and bone scintigraphy. From 3 to 5 years post-surgery, these examinations are conducted annually. Head MRI and bone scintigraphy may be skipped, considering patients’ age and tumour stage.

Locoregional recurrence was defined as a tumour relapse in the ipsilateral hilar and mediastinal lymph nodes, contralateral hilar, and mediastinal lymph nodes, in the ipsilateral thorax, including the resection margin of the lung or bronchus, and pleural dissemination and malignant pleural effusion. All other sites of recurrence, including the supraclavicular lymph nodes, were considered distant recurrence. Patients confirmed to have LRR and distant recurrence simultaneously were considered LRR. FLRR was defined as the period from the date of surgery to the first LRR, with censoring at the last follow-up date or occurrence of distant recurrence. RFS was defined as the period from the date of surgery to recurrence or death from any cause. OS was defined as the period from the date of surgery to death from any cause.

### Statistical analysis

Baseline data are expressed as medians and percentages for continuous and categorical variables, respectively. Continuous variables were compared using the 2-sided Wilcoxon rank-sum test, and categorical variables were analysed using the *χ*^2^ test. LRR-free survival was estimated using the Kaplan-Meier method and the associated hazard ratio (HR) and 95% CI were estimated using univariable Cox proportional hazards models. The cumulative incidences of LRR and the associated ratios were estimated using the univariable Fine-Gray subdistribution hazard model, treating death without recurrence and distant recurrence as competing events. In this study, we did not conduct statistical comparisons between the groups because the number of patients was small and the backgrounds were different to compare. HRs and 95% CIs were reported without *P-*values. All statistical analyses were conducted using the R 4.5.0 software (R Foundation for Statistical Computing, Vienna, Austria).

## RESULTS

### Overview of the cohort

**Figure 1.**
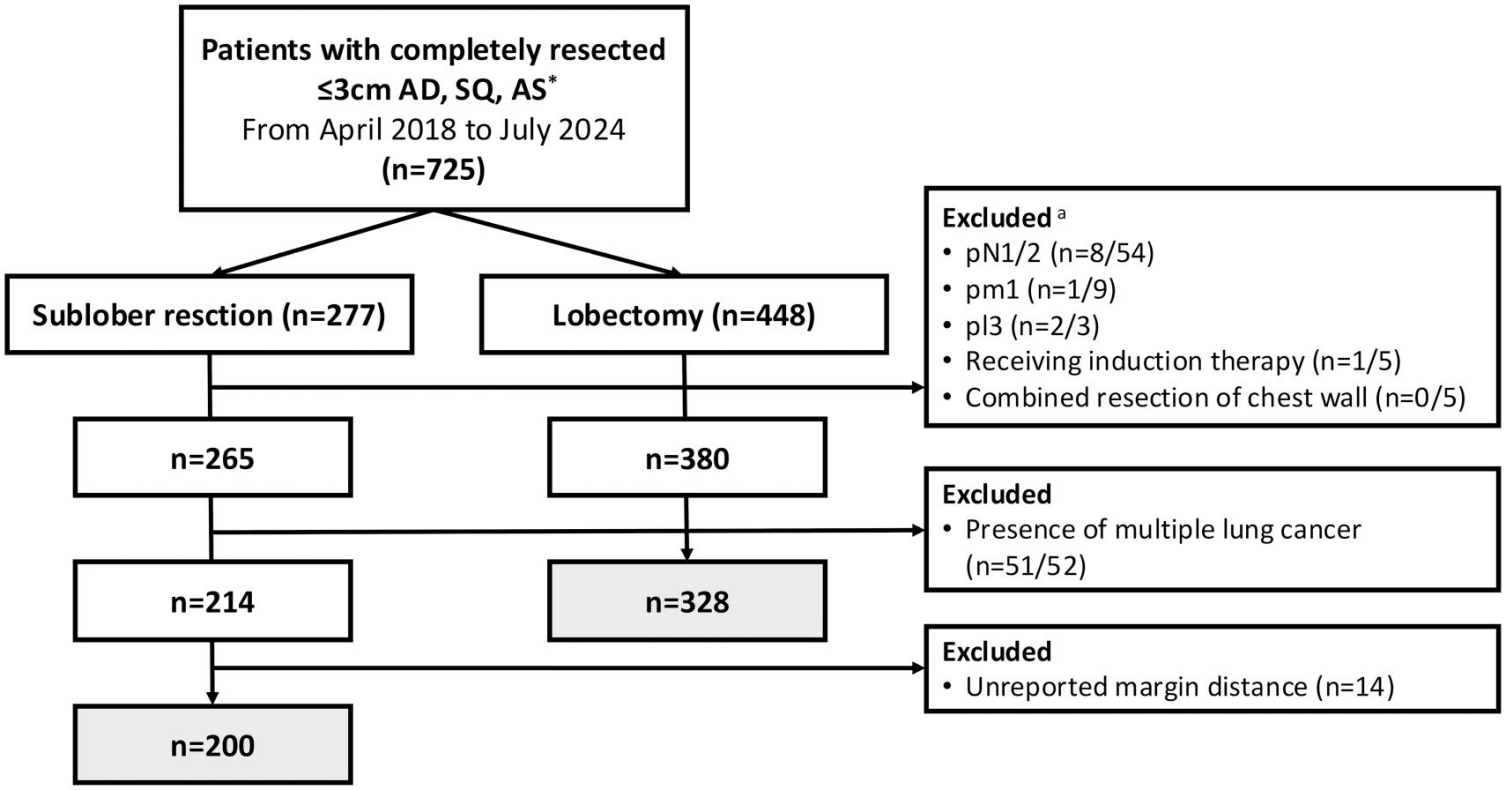
Flowchart of Study Cohort Selection. Abbreviations: AD, adenocarcinoma; AS, adenosquamous cell carcinoma; SQ, squamous cell carcinoma. ^a^Numbers do not add up, as patients can have more than one exclusion criterion.

In total, 725 patients underwent complete resection of pathological ≤3 cm adenocarcinoma, squamous cell carcinoma, and adenosquamous cell carcinoma between April 2018 and July 2024. Among them, 200 patients who underwent sublobar resection and 328 patients who underwent lobectomy were eligible, after excluding 197 patients who received induction therapy, underwent combined resection of chest wall, had interlobular pl3 tumours, had pathologically confirmed lymph node metastasis or intrapulmonary metastasis, had multiple lung cancers or with margin distance unreported (**Figure 1**). **Table 1** summarizes the clinical characteristics of the patients. The proportion of male sex, pathologically advanced stages, and high metabolic tumours (an SUVmax of >2.5) was higher in patients who underwent lobectomy. The tumour localization and radiological patterns were also different between the 2 groups.

**Table 1.** Clinicopathological Characteristics of the Overall Cohort

Characteristics	Lobectomy (*n* = 328)	Sublobar resection (*n* = 200)	SMD
Median age, years (IQR)	73 (68, 77)	72 (66, 78)	0.04
Sex			0.19
Male	186 (56.7)	95 (47.5)	
Female	142 (43.3)	105 (52.5)	
Pathological stage			0.91
Carcinoma in situ	11 (3.4)	33 (16.5)	
IA1	71 (21.6)	86 (43.0)	
IA2	121 (36.9)	62 (31.0)	
IA3	88 (26.8)	10 (5.0)	
IB	37 (11.3)	9 (4.5)	
Histology			0.13
Adenocarcinoma	285 (86.9)	182 (91.0)	
Squamous	39 (11.9)	16 (8.0)	
Adenosquamous	4 (1.2)	2 (1.0)	
Tumour location			0.88
Right upper lobe	148 (45.1)	27 (13.5)	
Right middle lobe	24 (7.3)	7 (3.5)	
Right lower lobe	73 (22.3)	50 (25.0)	
Left upper lobe	47 (14.3)	80 (40.0)	
Left lower lobe	36 (11.0)	36 (18.0)	
SUVmax >2.5			0.85
Yes	178 (54.3)	34 (17.0)	
No	116 (35.4)	119 (59.5)	
Not measured	34 (10.4)	47 (23.5)	
Radiological appearance			0.56
Pure GGN	3 (0.9)	20 (10.0)	
Subsolid nodule	204 (62.2)	143 (71.5)	
Pure-solid nodule	121 (36.9)	37 (18.5)	

Values are *n* (%) unless otherwise indicated.

Abbreviations: GGN, ground-glass nodule; IQR, interquartile range; SMD, standardized mean difference; SUVmax, maximum standardized uptake value.

### Relationship between margin distance and LRR

**Figure 2.**
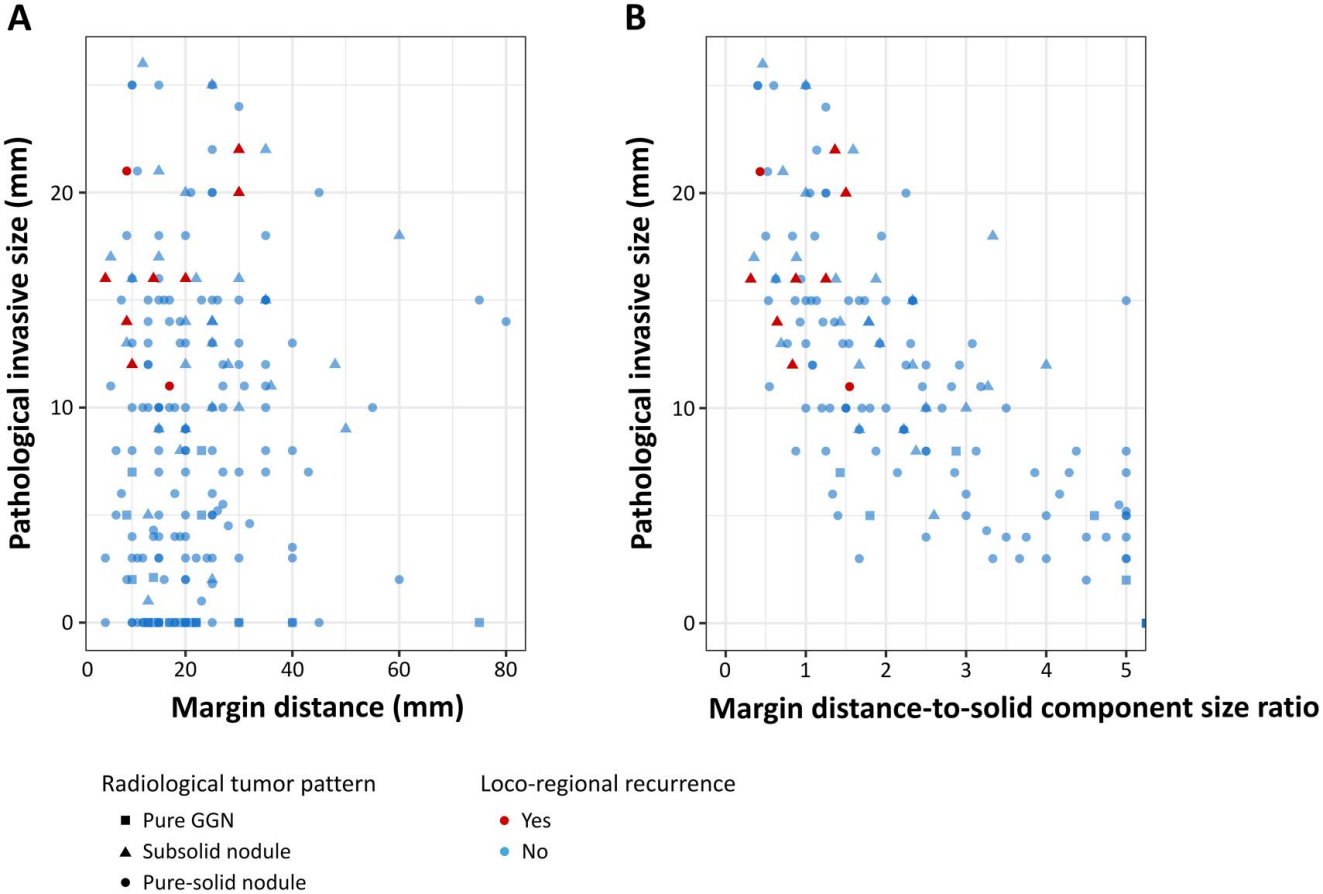
Scatter plots of the relationship between (A) margin distance and pathological invasive size and (B) margin distance-to-solid component size ratio and pathological invasive size in patients who underwent sublobar resection. A total of 22 cases were removed because they were outside the scale (margin distance-to-solid component size ratio was >5). There was no locoregional recurrence among these cases. Abbreviation: GGN, ground-glass nodule.

**Figure 2A** shows the scatter plots of the relationship between margin distance and pathological invasive size in patients who underwent sublobar resection. Cases with LRRs are represented as red dots. No patients with radiologically pure ground-glass nodules (GGNs), represented as square dots, had LRR. **Figure 2B** depicts the relationship between margin distance-to-solid component size ratio and the pathological invasive size. This cohort excluded patients with pathologically non-invasive nodules. All LRRs occurred in tumours with a margin distance of ≤30 mm or a margin distance-to-solid component size ratio of <2.0.

### Prognostic analysis compared with lobectomy

**Table 2** categorizes the patients into 3 groups: sufficient margin sublobar resection, insufficient margin sublobar resection, and lobectomy, after excluding 23 patients with pure GGNs. A total of 505 patients with 19 LRR events were included in the prognostic analysis. Sex, pathological stage, tumour location, and the proportion of high metabolic tumours were different between the 3 groups. The presence of lymphatic invasion and vascular invasion was also higher in patients who underwent lobectomy.

**Table 2.** Clinicopathological Characteristics After Excluding Patients With Radiologically Pure GGNs

	Sublobar resection (*n* = 180)		Lobectomy (*n* = 325)	
Characteristics	Insufficient margin (*n* = 32)	Sufficient margin (*n* = 148)		SMD^a^
Median age, years (IQR)	74 (70, 82)	72 (67, 77)	73 (68, 77)	0.25
Sex				0.40
Male	23 (71.9)	64 (43.2)	184 (56.6)	
Female	9 (28.1)	84 (56.8)	141 (43.4)	
Pathological stage				1.13
Carcinoma in situ	0 (0.0)	19 (12.8)	10 (3.1)	
IA1	2 (6.2)	78 (52.7)	70 (21.5)	
IA2	19 (59.4)	43 (29.1)	120 (36.9)	
IA3	6 (18.8)	4 (2.7)	88 (27.1)	
IB	5 (15.6)	4 (2.7)	37 (11.4)	
Histology				0.35
Adenocarcinoma	24 (75.0)	138 (93.2)	282 (86.8)	
Squamous	7 (21.9)	9 (6.1)	39 (12.0)	
Adenosquamous	1 (3.1)	1 (0.7)	4 (1.2)	
Tumour location				0.83
Right upper lobe	5 (15.6)	21 (14.2)	145 (44.6)	
Right middle lobe	0 (0.0)	4 (2.7)	24 (7.4)	
Right lower lobe	14 (43.8)	32 (21.6)	73 (22.5)	
Segment 6	8 (25.0)	16 (10.8)	31 (9.5)	
Basal segment	6 (18.8)	16 (10.8)	42 (12.9)	
Left upper lobe	6 (18.8)	66 (44.6)	47 (14.5)	
Upper division	5 (15.6)	57 (38.5)	36 (11.1)	
Lingula	1 (3.1)	9 (6.1)	11 (3.4)	
Left lower lobe	7 (21.9)	25 (16.9)	36 (11.1)	
Segment 6	1 (3.1)	15 (10.1)	10 (3.1)	
Basal segment	6 (18.8)	10 (6.8)	26 (8.0)	
SUVmax >2.5				0.67
Yes	11 (34.4)	22 (14.9)	177 (54.5)	
No	19 (59.4)	95 (64.2)	114 (35.1)	
Not measured	2 (6.2)	31 (20.9)	34 (10.5)	
Pleural invasion				0.32
pl0	27 (84.4)	144 (97.3)	285 (87.7)	
pl1	4 (12.5)	3 (2.0)	32 (9.8)	
pl2	1 (3.1)	1 (0.7)	7 (2.2)	
plX	0(0.0)	0(0.0)	1 (0.3)	
Lymphatic invasion				0.22
Ly0	31 (96.9)	143 (96.6)	287 (88.3)	
Ly1	1 (3.1)	5 (3.4)	38 (11.7)	
Vascular invasion				0.46
V0	32 (100.0)	138 (93.2)	268 (82.5)	
V1	0 (0.0)	10 (6.8)	57 (17.5)	
Details of sublobar resection				NA
Segmentectomy	21 (65.6)	118 (79.7)	NA	
Single-segment	14 (43.8)	78 (52.7)	NA	
Multi-segment	7 (21.9)	40 (27.0)	NA	
Wedge resection	11 (34.4)	30 (20.3)	NA	

Values are *n* (%) unless otherwise indicated.

a The reported SMD represents the average of the pairwise standardized mean differences across all group comparisons.

Abbreviations: GGN, ground-glass nodule; IQR, interquartile range; NA, not available; SMD, standardized mean difference; SUVmax, maximum standardized uptake value.

**Figure 3.**
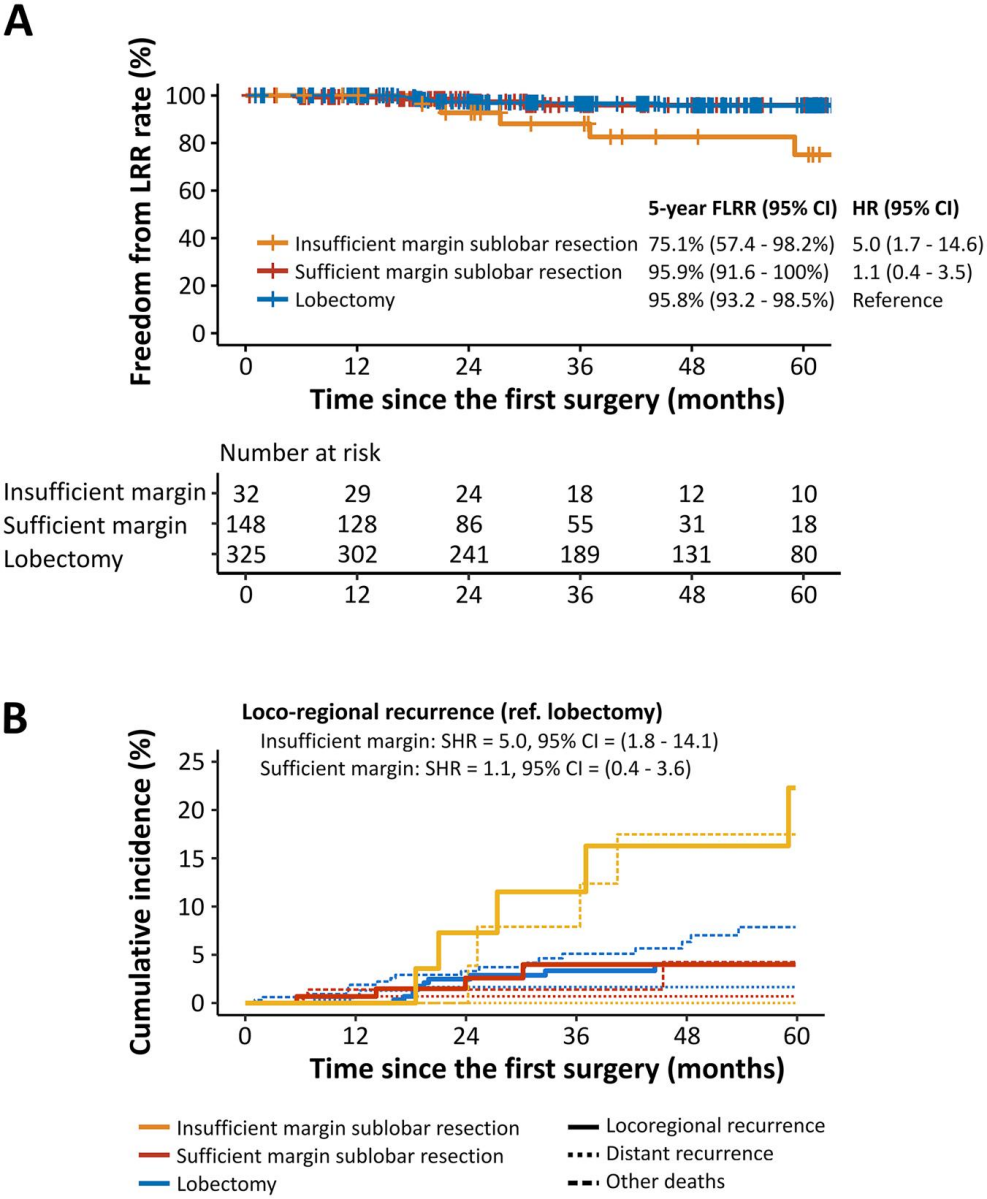
Prognosis of Patients in the Overall Cohort. (A) Kaplan-Meier diagram of freedom from LRR. (B) Cumulative incidence of LRR and other competing factors (death without recurrence and distant recurrence). Abbreviations: CI, confidence interval; FLRR, freedom from locoregional recurrence; HR, hazard ratio; LRR, locoregional recurrence; SHR, subdistribution hazard ratio.

In the overall cohort, the 5-year FLRR rate was 75.1% in the insufficient margin group, 95.9% in the sufficient margin group, and 95.8% in the lobectomy group, respectively. FLRR was worse in the insufficient group compared with the sufficient group (HR 4.5; 95% CI 1.2-16.9) or lobectomy group (HR 5.0; 95% CI 1.7-14.6), whereas FLRR in the sufficient margin group was similar to the lobectomy group (HR 1.1; 95% CI 0.4-3.5; **Figure 3A**). In the competing risk analysis, the risk of LRR was higher in the insufficient margin group compared to the sufficient margin group (subdistribution hazard ratio [SHR] 4.5; 95% CI 1.3-15.7) or lobectomy group (SHR 5.0; 95% CI 1.8-14.1), whereas the risk in the sufficient margin group was similar to the lobectomy group (SHR 1.1; 95% CI 0.4-3.6; **Figure 3B**). RFS and OS results are depicted in **Figure S1**. OS was also worse in the insufficient margin group than in the sufficient margin group (HR 3.3; 95% CI 1.1-9.4) or lobectomy group (HR 2.64; 95% CI 1.14-6.10).

**Figure 4.**
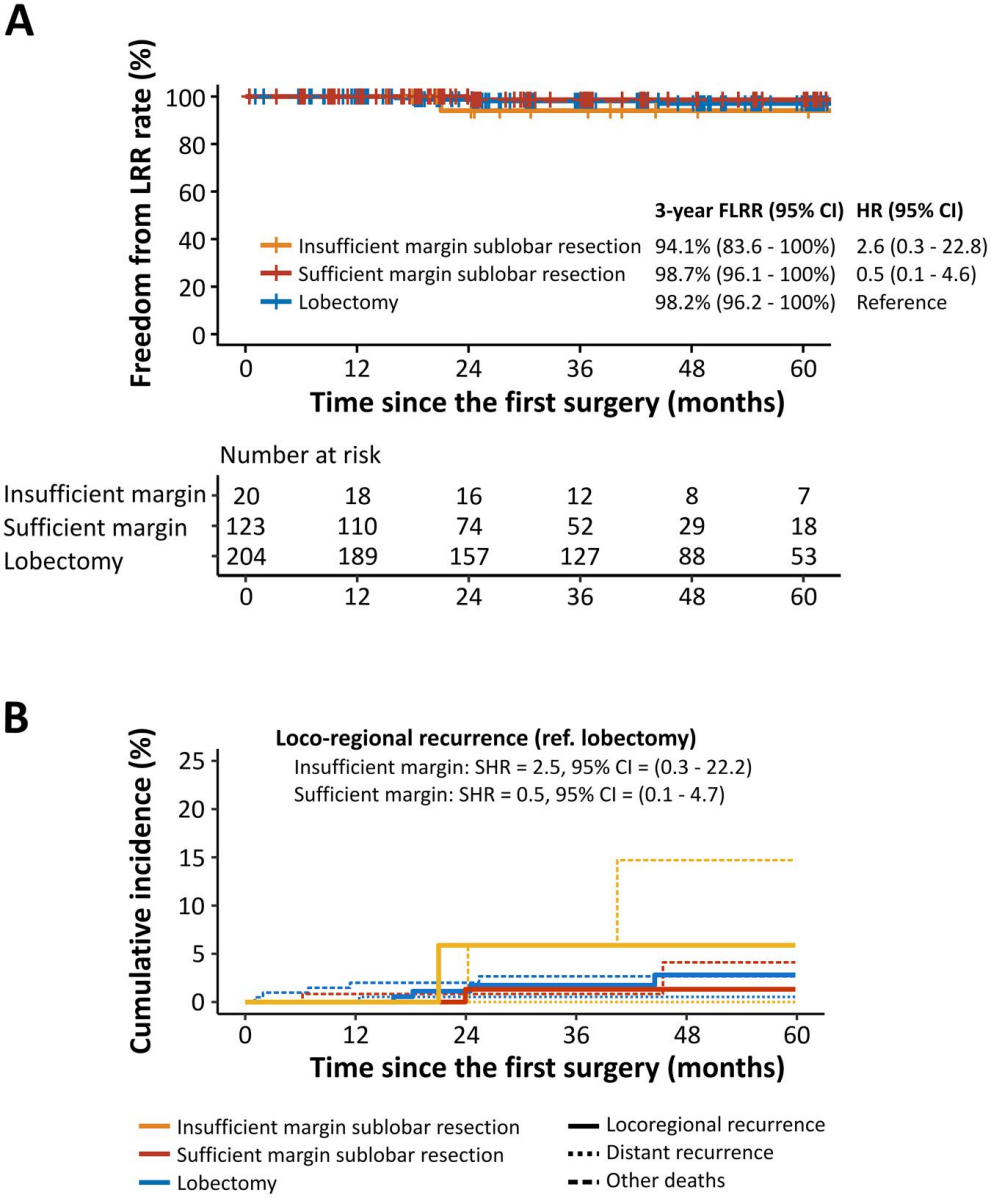
Prognosis of Patients in the Subsolid Nodule Cohort. (A) Kaplan-Meier diagram of freedom from LRR. (B) Cumulative incidence of LRR and other competing factors (death without recurrence and distant recurrence). Abbreviations: CI, confidence interval; FLRR, freedom from locoregional recurrence; HR, hazard ratio; LRR, locoregional recurrence; SHR, subdistribution hazard ratio.

**Figure 5.**
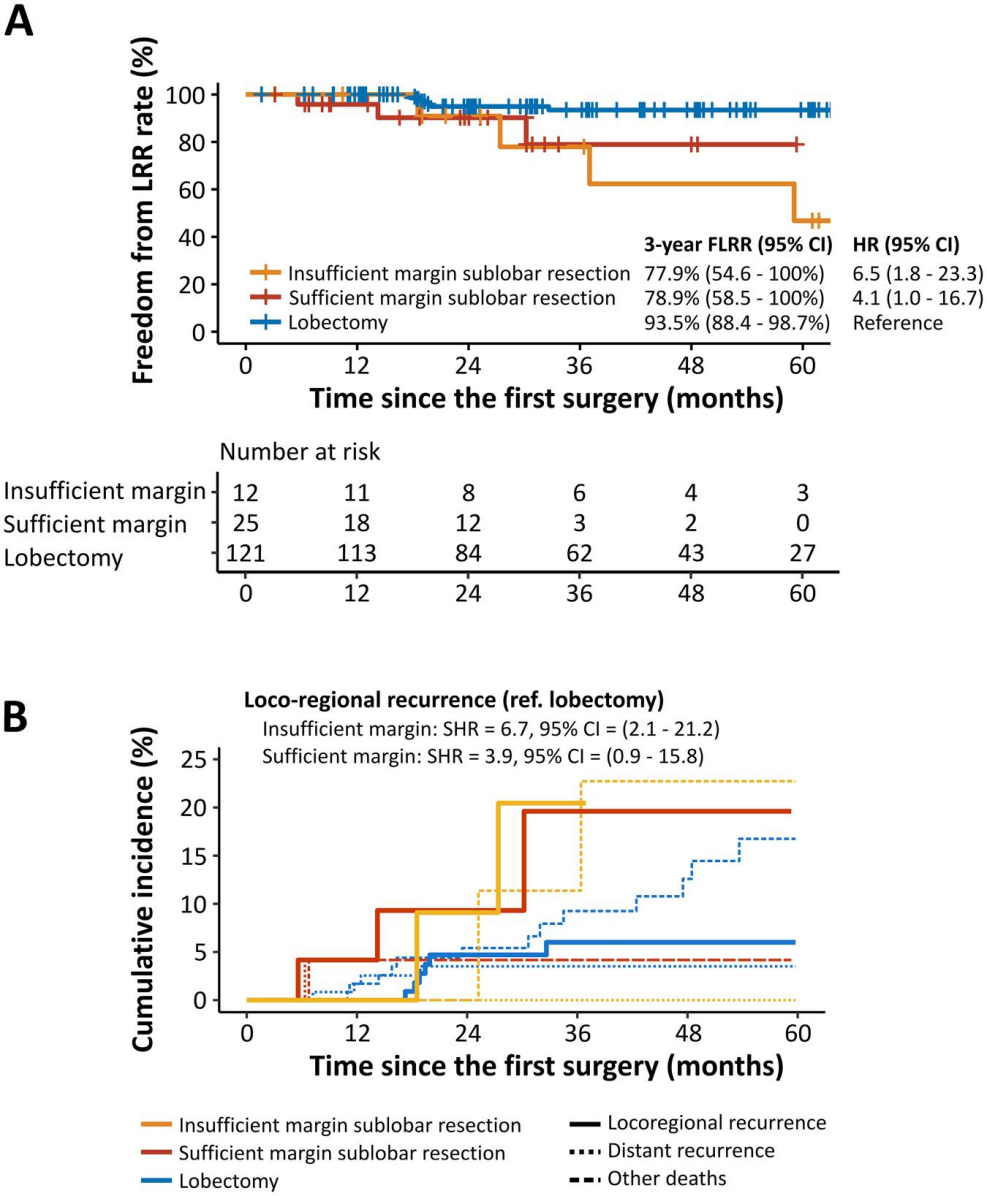
Prognosis of Patients in the Pure-Solid Nodule Cohort. (A) Kaplan-Meier diagram of freedom from LRR. (B) Cumulative incidence of LRR and other competing factors (death without recurrence and distant recurrence). Abbreviations: CI, confidence interval; FLRR, freedom from locoregional recurrence; HR, hazard ratio; LRR, locoregional recurrence; SHR, subdistribution hazard ratio.

**Figure 4** shows the results of FLRR and competing risk analysis in the subsolid nodule cohort. The results were similar to those ones in the overall cohort. **Figure 5** depicts the results of FLRR and competing risk analysis in the pure-solid nodule cohort. In contrast to the results of the overall cohort and the subsolid nodule cohort as mentioned earlier, the FLRR was worse in the sufficient margin group (HR 4.1; 95% CI 1.0-16.7) and the insufficient margin group (HR 6.6; 95% CI 1.8-23.3) than in the lobectomy group. In the competing risk analysis, the risk of LRR was higher in the sufficient margin group (SHR 3.9; 95% CI 0.9-15.8) as well as the insufficient group (SHR 6.7; 95% CI 2.1-21.2), compared to the lobectomy group.

Finally, we demonstrated the FLRR and the cumulative incidence of LRR between patients with conventional sufficient margins (at least 2 cm or the size of tumour) and those with margins with no LRR demonstrated in the study (at least 3 cm and a margin distance-to-solid component size ratio of ≥2.0) for reference (**Figure S2**).

### Influence of the type of sublobar resection on LRR

**Figure S3** depicts the comparison of FLRR and competing risk analysis grouped by the types of sublobar resection. These data showed that wedge resection had a higher risk of LRR. Moreover, when patients were classified into 4 groups considering the surgical margin, the risk of LRR was worse in the insufficient margin wedge resection group than in the other 3 groups.

### First site of recurrence

**Figure S4** displays the details of 25 patients who experienced recurrence, among whom 18 patients had pure-solid nodules and 7 patients had subsolid nodules. There were 18 adenocarcinomas and 7 squamous cell carcinomas. Among 19 patients who experienced LRR, 13 experienced only LRR and 6 patients experienced LRR and distant metastasis simultaneously. Patients who underwent sublobar resection for right lower lobe tumour were most likely to experience LRR (8.0%). The frequency of recurrence in other lobes is depicted in **Figure S4**.

### Treatment after recurrence

**Figure S5** depicts the treatment details after recurrence of 25 patients who experienced recurrence. Among the 13 patients with LRR, 5 received a treatment aiming for cure, including 2 patients for surgery, 1 patient for stereotactic radiotherapy, and 2 patients for chemoradiotherapy. Nevertheless, among patients who underwent surgery, one patient was found to have pleural dissemination and received systemic anticancer therapy, and the other experienced re-recurrence and was provided best supportive care. One patient treated with stereotactic radiotherapy progressed without recurrence but died due to a comorbidity. Among the 2 patients who received chemoradiotherapy, one patient achieved long-term recurrence-free survival, and the other could not achieve complete response and continued to receive systemic anticancer therapy. One patient with distant metastasis received stereotactic radiotherapy for a contralateral pulmonary nodule as an oligometastasis and progressed without recurrence.

## DISCUSSION

This study suggested that patients who underwent sublobar resection with a sufficient margin (more than the invasive size of the nodule) achieved better local control than those with an insufficient margin, and sublobar resection with a sufficient margin was comparable to lobectomy. Conversely, in the small-sized subgroup analysis, which categorized the tumours into subsolid and pure-solid nodules, pure-solid nodules showed the opposite results. In the pure-solid nodule cohort, even the sufficient margin sublobar resection group tended to have more LRRs than the lobectomy group, although patients with subsolid nodules attained the same result as the overall cohort. Considering the presence of imbalance in baseline clinicopathological characteristics, these findings, derived from univariable analyses, should be interpreted with caution and regarded as predictive rather than causal.

Two randomized controlled trials, JCOG0802 and CALGB 140503, demonstrated that the LRR rate was higher in sublobar resection than in lobectomy.^1^^,^^2^ Our results are inconsistent with these findings. Although our results may have been influenced by imbalances in baseline clinicopathological characteristics, which could partially explain this inconsistency, another potential reason is that those trials included insufficient margin cases. Although both studies recommended securing a surgical margin greater than the maximum tumour diameter or 20 mm, JCOG0802 included 18.6% of patients with a margin distance less than the tumour size^6^ and CALGB 140503 included 38.2% of patients in the wedge resection group and 22.7% of patients in the segmentectomy group with a margin/tumour ratio of <1.^7^ A supplemental study of JCOG0802 demonstrated that a margin distance less than the tumour size was significantly associated with LRR in the multivariable analysis.^6^

This risk of increased LRR rates due to insufficient margins has been known for some time. For instance, Schuchert et al reported that a tumour diameter ratio of <1 was a significant independent predictor of recurrence following resection of clinical stage I NSCLC.^8^ Akamine et al demonstrated that segmentectomy is a feasible procedure compared with wedge resection, particularly for clinical stage IA2 NSCLC, possibly due to the small proportion of adequate surgical margins in the wedge resection group.^9^ Similarly, our study has indicated that patients underwent wedge resection, especially the insufficient margin wedge resection have higher risk of LRR. A crucial factor in performing sublobar resection is determining whether a sufficient margin can be secured. We must also pay even more attention when performing wedge resection.

Regarding the adequate margin distance, several studies have attempted to clarify this issue. For instance, Kamtam et al concluded that a margin distance-to-solid component size ratio of >1.0 may be a more reliable factor than margin distance alone to minimize local recurrence.^4^ Moreover, Huang et al reported that a margin distance of ≥20 mm decreased the LRR and improved the survival outcome.^5^ These results were eventually the same as the conventional cut-off value. Nevertheless, to validate the threshold, these studies compared the prognosis between the sufficient margin group and insufficient margin group, without comparing with the conventional gold standard lobectomy. We demonstrated the comparable outcome of sublobar resection with a sufficient margin with that of lobectomy.

Regarding pure-solid nodules, our study indicated that even the sufficient margin sublobar resection group had more LRRs than the lobectomy group. This result suggests that a margin of the invasive size of the nodule might be insufficient for pure-solid tumours. This finding is consistent with several previous reports indicating that LRR occurs more frequently after segmentectomy in patients with radiologically pure-solid NSCLC.^6^^,^^10^ Nonetheless, it remains unclear whether sublobar resection is inadequate or whether the ordinary margin distance is insufficient for pure-solid tumours. The scatter plot of the relationship between margin distance and pathological invasive size revealed that no LRR occurred in nodules that secured a margin distance-to-solid component size ratio of >2.0. It is ambiguous whether a margin distance-to-solid component size ratio of >2.0 is sufficient for pure-solid nodules or whether this result is due to the small sample size.

Regarding the prognosis after recurrence, previous randomized studies have demonstrated that a higher frequency of local recurrence in patients with sublobar resection did not affect OS.^1^^,^^2^ Nevertheless, our study indicated the opposite results, wherein the OS in the insufficient margin group was poorer than in the lobectomy and sufficient margin groups. A potential reason was that only a few cases with LRR received local control treatments such as surgery and radiotherapy aiming for a cure. The presence of high-risk patients who underwent sublobar resection as a compromised surgery and LRR cases that are not candidates for local treatment, such as multiple lymph node metastases or pleural dissemination, may have contributed to this result.

This study has several limitations. First, this was a small-sized, retrospective, single-institution study. The 3 groups differed in baseline characteristics; especially, the selection bias of a greater proportion of patients with advanced stage in the lobectomy group was a major concern. This could result in an overestimation of prognosis in the sublobar resection group. Furthermore, because of the small sample size, we could not apply any specific methods to adjust the backgrounds. Hence, we did not perform statistical comparisons between the groups, and the analyses should be considered exploratory. Second, only a few cases were evaluated regarding the spread through air space (STAS) in this study. Tumours with STAS have a higher risk of LRR, and the indication of sublobar resection for these cases must be stricter.^11^^,^^12^ Third, we measured a surgical margin by using a fixed specimen, which could be slightly different from the *in vivo* condition and may introduce an informative bias. Finally, the follow-up duration was relatively short. Local recurrence, particularly cut-end recurrence, occasionally occurs beyond 5 years after sublobar resection.^13^^,^^14^ Therefore, a sufficient follow-up period is necessary to obtain more precise results.

## CONCLUSION

This study suggested patients who underwent sublobar resection with a sufficient margin (more than the invasive size of the nodule) achieved better local control than those with an insufficient margin, and sublobar resection with a sufficient margin was comparable to lobectomy. Conversely, for pure-solid nodules, the conventional margin distance did not appear to be sufficient. An adequate margin distance might be different depending on the radiological patterns of the tumour.

## Supplementary Material

ivag045_Supplementary_Data

## Data Availability

The data underlying this article will be shared on reasonable request to the corresponding author.
