# Supplementary material for: Sublobar Resection With Adequate Margin is Comparable to Lobectomy in Locoregional Recurrence
Source: Interdiscip Cardiovasc Thorac Surg. 2026 Feb 10;41(2):ivag045. doi: 10.1093/icvts/ivag045 (PMC12953239; doi:10.1093/icvts/ivag045)
Supplement: ivag045_Supplementary_Data [file ivag045_supplementary_data.zip › FigureS5.pdf]

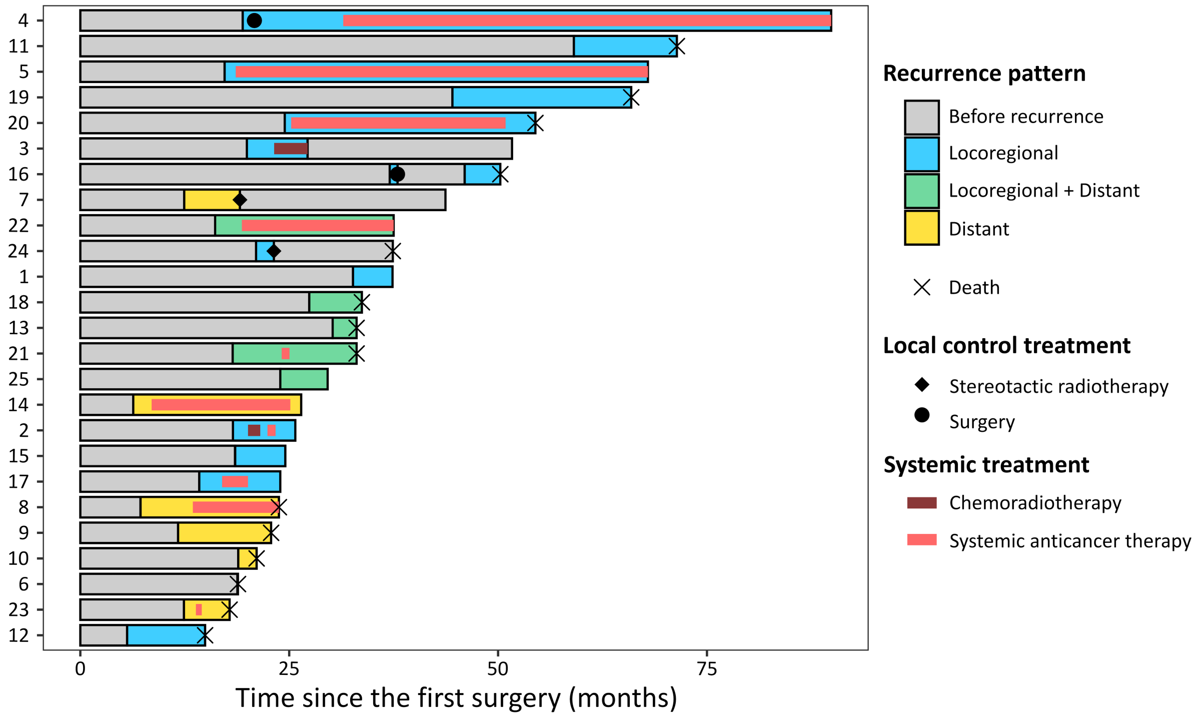

Figure S5. Swimmer's plot showing the treatment after recurrence.
Bar length indicates the duration of follow-up shown in months after the first surgery.
