# Supplementary material for: Sublobar Resection With Adequate Margin is Comparable to Lobectomy in Locoregional Recurrence
Source: Interdiscip Cardiovasc Thorac Surg. 2026 Feb 10;41(2):ivag045. doi: 10.1093/icvts/ivag045 (PMC12953239; doi:10.1093/icvts/ivag045)
Supplement: ivag045_Supplementary_Data [file ivag045_supplementary_data.zip › FigureS1.pdf]

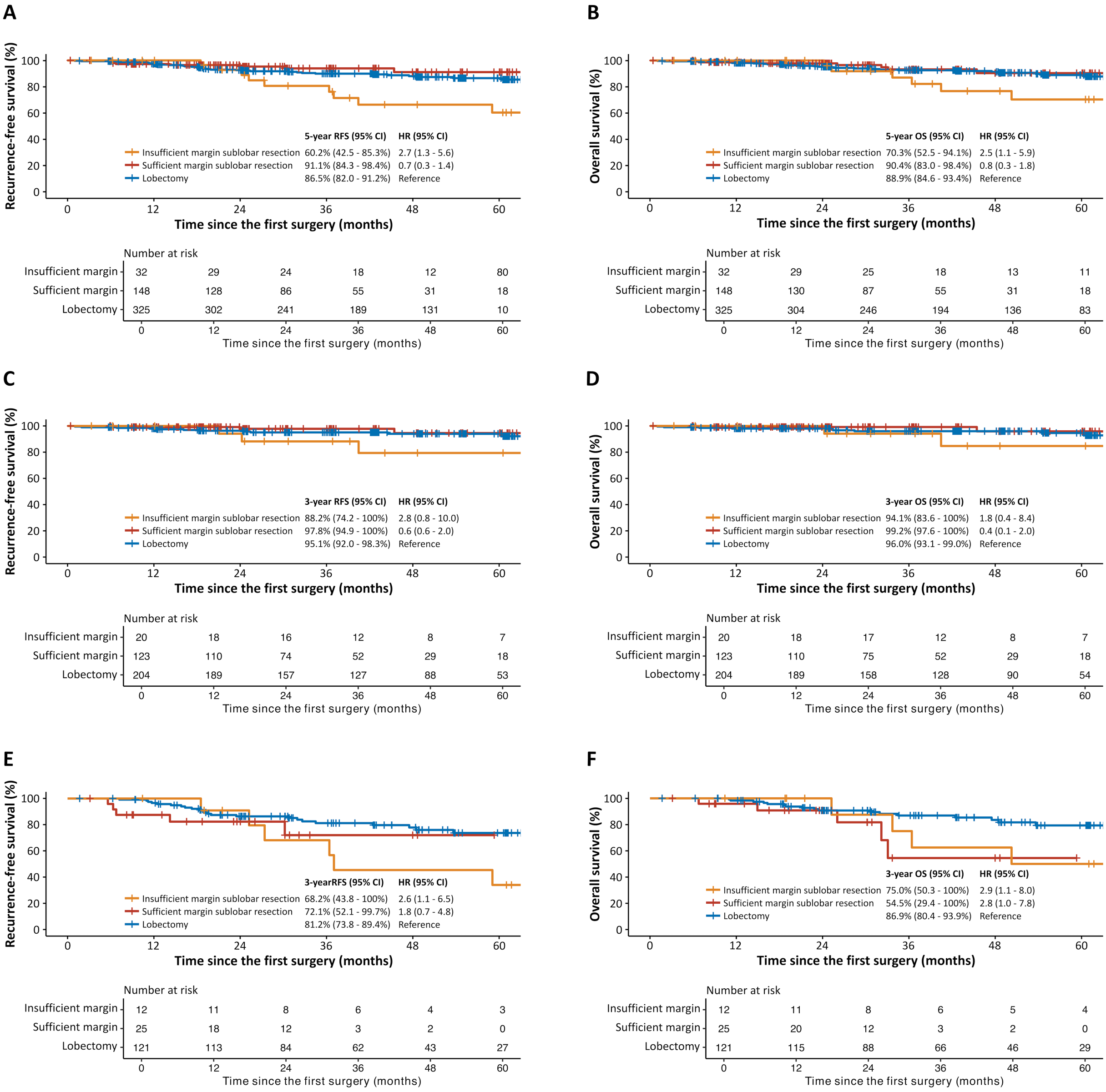

Figure S1. Recurrence-free survival (A, C, E) and overall survival (B, D, F) of patients in the overall cohort (A, B), subsolid nodule cohort (C, D) and solid nodule cohort (E, F). CI: confidence interval; HR: hazard ratio; HR: hazard ratio; RFS: recurrence-free survival; OS: overall survival.
