# Supplementary material for: Sublobar Resection With Adequate Margin is Comparable to Lobectomy in Locoregional Recurrence
Source: Interdiscip Cardiovasc Thorac Surg. 2026 Feb 10;41(2):ivag045. doi: 10.1093/icvts/ivag045 (PMC12953239; doi:10.1093/icvts/ivag045)
Supplement: ivag045_Supplementary_Data [file ivag045_supplementary_data.zip › FigureS2.pdf]

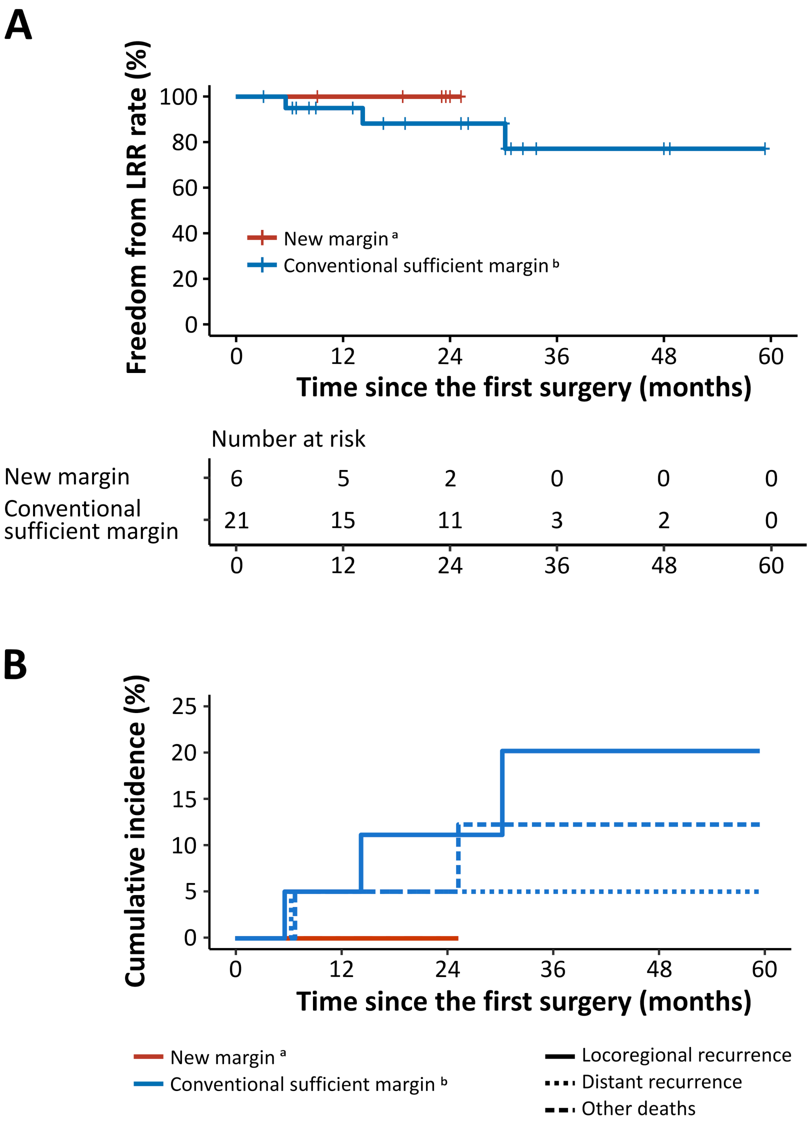

Figure S2. Prognosis of patients categorised by margin distance in the overall cohort. (A) Kaplan–Meier diagram of freedom from LRR. (B) Cumulative incidence of LRR and other competing factors (death without recurrence and distant recurrence).

[a] new margin: at least 3cm and a margin distance-to-solid component size ratio of ≥2.0

[b] conventional sufficient margin: at least 2cm or the size of tumour

LRR: locoregional recurrence
