# Supplementary material for: Sublobar Resection With Adequate Margin is Comparable to Lobectomy in Locoregional Recurrence
Source: Interdiscip Cardiovasc Thorac Surg. 2026 Feb 10;41(2):ivag045. doi: 10.1093/icvts/ivag045 (PMC12953239; doi:10.1093/icvts/ivag045)
Supplement: ivag045_Supplementary_Data [file ivag045_supplementary_data.zip › FigureS3.pdf]

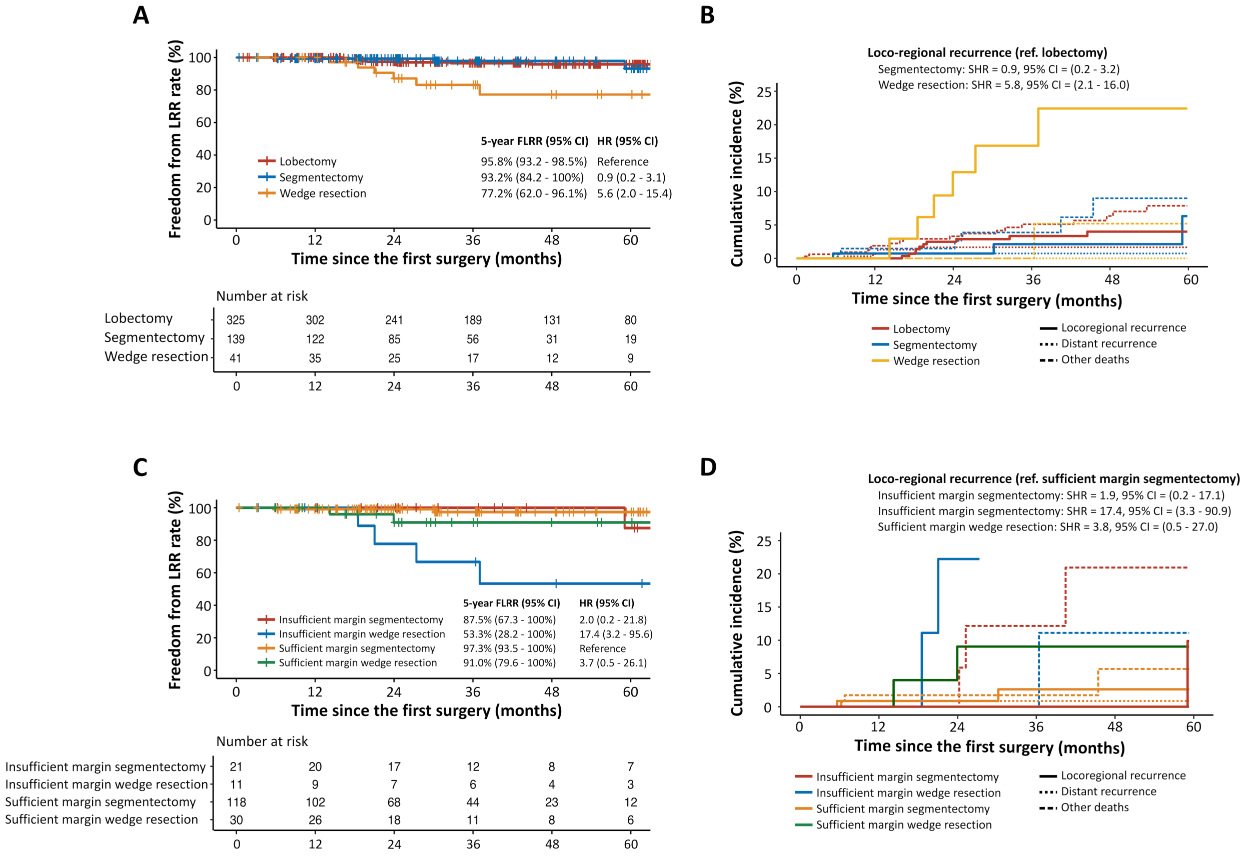

Figure S3. Prognosis of patients categorised by the type of surgery in the overall cohort. (A, C) Kaplan–Meier diagram of freedom from LRR.
(B, D) Cumulative incidence of LRR and other competing factors (death without recurrence and distant recurrence).
CI: confidence interval; FLRR: freedom from locoregional recurrence; HR: hazard ratio; LRR: locoregional recurrence; SHR: subdistribution hazard ratio.
