# Supplementary figures and images for: Sublobar Resection With Adequate Margin is Comparable to Lobectomy in Locoregional Recurrence
Source: Interdiscip Cardiovasc Thorac Surg. 2026 Feb 10;41(2):ivag045. doi: 10.1093/icvts/ivag045 (PMC12953239; doi:10.1093/icvts/ivag045)

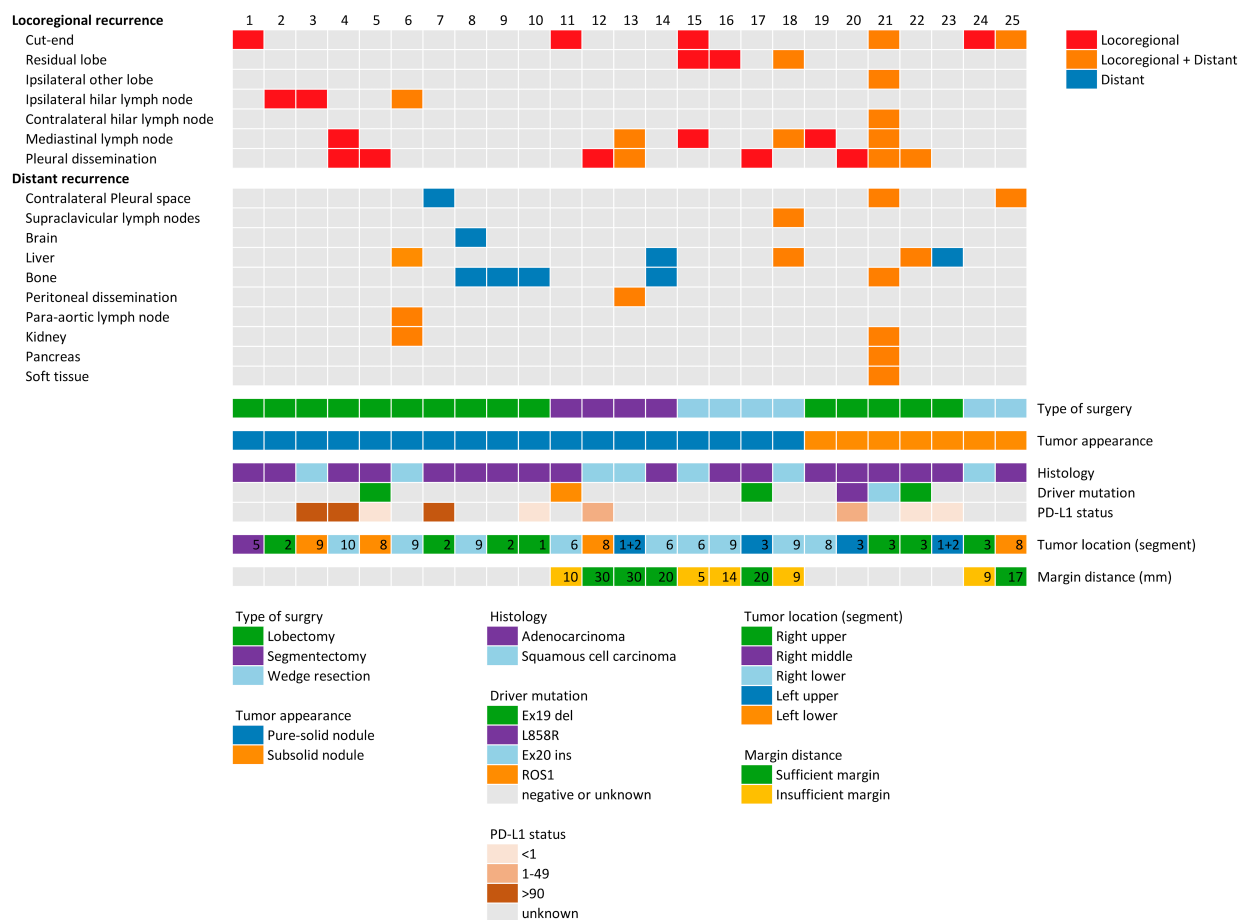

Figure S1. Recurrence site profiles of 25 patients who experienced recurrence.

Supplement: ivag045_Supplementary_Data [file ivag045_supplementary_data.zip › FigureS4.pdf]
